# Supplementary material for: MetaRibo-Seq measures translation in microbiomes
Source: Nat Commun. 2020 Jun 29;11:3268. doi: 10.1038/s41467-020-17081-z (PMC7324362; doi:10.1038/s41467-020-17081-z)
Supplement: Supplementary file 10 — Supplementary Data 7 [file 41467_2020_17081_MOESM10_ESM.zip › File2/Confidence_High_Taxonomy/275488_out.krona.html]

Javascript must be enabled to view this page.

members
magnitude
magnitudeUnassigned
count
unassigned
taxon
rank

275488\_out

283

2
superkingdom
283

phylum
976
283

class
200643
283

order
171549
1
283

SRS892882\_contig\_number\_13294

family
2005519
1

1
genus
397864

1

SRS050801\_contig\_number\_3039
397865
species

4
family
171552

2
genus
838

1

SRS049995\_contig\_number\_19712
1262925
species


SRS893366\_contig\_number\_19776
165179
species
1

1
genus
577309

species
454154

SRS020328\_contig\_number\_11954
1

species
370804

SRS023604\_contig\_number\_1166
1

104
815
family

genus
816

SRS018427\_contig\_number\_9493SRS019787\_contig\_number\_13791SRS021219\_contig\_number\_contig-100\_579.68437SRS1041133\_contig\_number\_9596SRS1041143\_contig\_number\_contig-100\_1192.30984SRS1041144\_contig\_number\_8119SRS1055076\_contig\_number\_12534SRS148196\_contig\_number\_contig-100\_39.247238
104
8

28

SRS013098\_contig\_number\_48089SRS013800\_contig\_number\_4732SRS019445\_contig\_number\_36676SRS019496\_contig\_number\_contig-100\_668.113558SRS019685\_contig\_number\_6724SRS023583\_contig\_number\_16157SRS023715\_contig\_number\_contig-100\_234.176009SRS024435\_contig\_number\_14329SRS042284\_contig\_number\_7018SRS049900\_contig\_number\_1020SRS056259\_contig\_number\_23882SRS063370\_contig\_number\_10440SRS064645\_contig\_number\_1982SRS075821\_contig\_number\_contig-100\_1403.126698SRS077294\_contig\_number\_2367SRS100021\_contig\_number\_6195SRS1041031\_contig\_number\_16047SRS1041129\_contig\_number\_15549SRS104693\_contig\_number\_contig-100\_936.92610SRS1054716\_contig\_number\_5177SRS147088\_contig\_number\_3929SRS149181\_contig\_number\_1118SRS893230\_contig\_number\_2608SRS893252\_contig\_number\_466SRS893270\_contig\_number\_24410SRS893292\_contig\_number\_8894SRS893295\_contig\_number\_7335SRS893300\_contig\_number\_contig-100\_26.79480
338188
species


SRS015782\_contig\_number\_contig-100\_435.254951SRS016132\_contig\_number\_4704SRS018888\_contig\_number\_9775SRS019161\_contig\_number\_1079SRS021948\_contig\_number\_1944SRS044535\_contig\_number\_13130SRS047044\_contig\_number\_18292SRS051882\_contig\_number\_15547SRS076804\_contig\_number\_18610SRS077231\_contig\_number\_23280SRS104036\_contig\_number\_contig-100\_290.62284SRS1041138\_contig\_number\_11599SRS104311\_contig\_number\_10805SRS143876\_contig\_number\_contig-100\_896.228550SRS144506\_contig\_number\_11500SRS146764\_contig\_number\_33405
species
817
16

1796613
species

SRS020328\_contig\_number\_34865
1

1
species
457387

SRS058070\_contig\_number\_31436

28111
species

SRS143780\_contig\_number\_14646
1

2

SRS023971\_contig\_number\_2069SRS046068\_contig\_number\_911
species
1263045

1

SRS017701\_contig\_number\_17309
species
47678

4

SRS014235\_contig\_number\_40112SRS043411\_contig\_number\_contig-100\_182.109094SRS143523\_contig\_number\_23352SRS147653\_contig\_number\_6899
species
821

species
2292935

SRS064232\_contig\_number\_2804
1

1
674529
species

SRS143722\_contig\_number\_7709

4
species
28116

SRS013382\_contig\_number\_1444SRS051882\_contig\_number\_5408SRS101376\_contig\_number\_29500SRS143876\_contig\_number\_30583

1
species
2292927

SRS013638\_contig\_number\_19838

species
1739298

SRS148159\_contig\_number\_54202
1

species
818

SRS013951\_contig\_number\_34080SRS1041095\_contig\_number\_contig-100\_1172.35269SRS144753\_contig\_number\_4234SRS147425\_contig\_number\_11612
4


SRS016095\_contig\_number\_24326SRS020869\_contig\_number\_28780SRS048164\_contig\_number\_2250SRS049959\_contig\_number\_27369SRS1041133\_contig\_number\_20330SRS144537\_contig\_number\_46473
species
246787
6

4
820
species

SRS013940\_contig\_number\_21908SRS042284\_contig\_number\_5701SRS075716\_contig\_number\_3980SRS098717\_contig\_number\_15649


SRS018133\_contig\_number\_13115SRS058770\_contig\_number\_18021
2292928
species
2

3

SRS045004\_contig\_number\_14671SRS076929\_contig\_number\_26919SRS1041140\_contig\_number\_contig-100\_526.124045
329854
species

1

SRS1041036\_contig\_number\_653
2292941
species

1
310297
species

SRS019381\_contig\_number\_5614

species
357276

SRS017622\_contig\_number\_620
1

7
46506
species

SRS016954\_contig\_number\_24946SRS024625\_contig\_number\_18088SRS042966\_contig\_number\_19013SRS043768\_contig\_number\_18898SRS074670\_contig\_number\_4122SRS097889\_contig\_number\_contig-100\_1055.217180SRS147271\_contig\_number\_14885

1
species
2292931

SRS142781\_contig\_number\_10180

4
2292944
species

SRS014459\_contig\_number\_41116SRS042966\_contig\_number\_12269SRS050941\_contig\_number\_12886SRS1041037\_contig\_number\_contig-100\_1849.84098

1853231
family
16

3
genus
574697

species
2093856

SRS024132\_contig\_number\_25
1


SRS019397\_contig\_number\_5983SRS058770\_contig\_number\_9242
species
544645
2

13
283168
genus

626933
species

SRS014235\_contig\_number\_16229SRS019685\_contig\_number\_12523SRS024132\_contig\_number\_30636SRS053335\_contig\_number\_11012SRS077024\_contig\_number\_3921SRS078419\_contig\_number\_17606SRS1041133\_contig\_number\_28164SRS104636\_contig\_number\_23759SRS147766\_contig\_number\_17620SRS149784\_contig\_number\_21742SRS893279\_contig\_number\_15329
11

2
28118
species

SRS024132\_contig\_number\_2092SRS052697\_contig\_number\_34478

171550
family
144

239759
genus
6
144

SRS014923\_contig\_number\_41840SRS016495\_contig\_number\_contig-100\_686.122484SRS019968\_contig\_number\_14678SRS049995\_contig\_number\_4390SRS065504\_contig\_number\_30802SRS1054928\_contig\_number\_contig-100\_592.51175

1
species
1118061

SRS147766\_contig\_number\_13338

1

SRS098571\_contig\_number\_28821
species
1896970

species
2292911

SRS017821\_contig\_number\_contig-100\_1060.192261SRS065504\_contig\_number\_34683SRS077024\_contig\_number\_contig-100\_645.104822SRS144537\_contig\_number\_42553SRS148721\_contig\_number\_39122
5

1263035
species

SRS011084\_contig\_number\_25716SRS012969\_contig\_number\_21034SRS014855\_contig\_number\_17621SRS054905\_contig\_number\_16224
4

species
1872444

SRS053335\_contig\_number\_15130SRS142503\_contig\_number\_46559SRS144183\_contig\_number\_40831SRS148091\_contig\_number\_2431SRS150029\_contig\_number\_19926
5

1

SRS011239\_contig\_number\_18665
1499682
species

5

SRS015264\_contig\_number\_23070SRS050299\_contig\_number\_contig-100\_892.78229SRS063190\_contig\_number\_contig-100\_1301.95062SRS893259\_contig\_number\_contig-100\_1295.51219SRS971276\_contig\_number\_19511
species
1896969


SRS017916\_contig\_number\_13176SRS024435\_contig\_number\_25962SRS049959\_contig\_number\_45557SRS098644\_contig\_number\_49631
328814
species
4

2
species
328813

SRS058770\_contig\_number\_24461SRS148424\_contig\_number\_26318

15
28117
species

SRS011302\_contig\_number\_23528SRS013476\_contig\_number\_38666SRS016897\_contig\_number\_1741SRS017191\_contig\_number\_16568SRS018836\_contig\_number\_4291SRS019267\_contig\_number\_3381SRS022137\_contig\_number\_5616SRS053649\_contig\_number\_22409SRS054059\_contig\_number\_contig-100\_304.49007SRS104036\_contig\_number\_8387SRS1041032\_contig\_number\_9854SRS1041033\_contig\_number\_26144SRS1041091\_contig\_number\_10360SRS143342\_contig\_number\_17686SRS971276\_contig\_number\_11459


SRS011084\_contig\_number\_31821SRS012273\_contig\_number\_39929SRS013215\_contig\_number\_contig-100\_1031.22912SRS013476\_contig\_number\_42143SRS013951\_contig\_number\_16616SRS013965\_contig\_number\_contig-100\_675.229901SRS014287\_contig\_number\_18546SRS014613\_contig\_number\_22292SRS014683\_contig\_number\_9045SRS014948\_contig\_number\_16273SRS015065\_contig\_number\_31629SRS015190\_contig\_number\_16152SRS015217\_contig\_number\_contig-100\_327.185557SRS015431\_contig\_number\_24850SRS015694\_contig\_number\_24828SRS015782\_contig\_number\_43757SRS015794\_contig\_number\_7869SRS015854\_contig\_number\_20119SRS015890\_contig\_number\_20425SRS016095\_contig\_number\_29436SRS016203\_contig\_number\_13216SRS016267\_contig\_number\_contig-100\_738.87747SRS016335\_contig\_number\_37691SRS017103\_contig\_number\_41828SRS017247\_contig\_number\_10649SRS018541\_contig\_number\_9489SRS018817\_contig\_number\_18905SRS018888\_contig\_number\_4632SRS018984\_contig\_number\_16259SRS019068\_contig\_number\_87107SRS019161\_contig\_number\_contig-100\_239.242522SRS019267\_contig\_number\_contig-100\_809.61650SRS019601\_contig\_number\_27054SRS019685\_contig\_number\_22680SRS020394\_contig\_number\_17577SRS022071\_contig\_number\_38933SRS024075\_contig\_number\_14277SRS024132\_contig\_number\_41807SRS024388\_contig\_number\_14801SRS045004\_contig\_number\_14609SRS045645\_contig\_number\_25509SRS049402\_contig\_number\_contig-100\_541.117263SRS049773\_contig\_number\_35971SRS049900\_contig\_number\_19090SRS051610\_contig\_number\_11070SRS056519\_contig\_number\_17185SRS056537\_contig\_number\_56258SRS057480\_contig\_number\_contig-100\_211.80871SRS063040\_contig\_number\_42022SRS063518\_contig\_number\_38853SRS063813\_contig\_number\_contig-100\_1118.58349SRS064276\_contig\_number\_19573SRS075773\_contig\_number\_34648SRS075984\_contig\_number\_25763SRS076929\_contig\_number\_contig-100\_389.148215SRS077127\_contig\_number\_14946SRS078419\_contig\_number\_21537SRS098620\_contig\_number\_2843SRS098655\_contig\_number\_contig-100\_234.89451SRS098827\_contig\_number\_8842SRS098881\_contig\_number\_26377SRS101376\_contig\_number\_22780SRS1041132\_contig\_number\_contig-100\_1014.62995SRS1041134\_contig\_number\_13391SRS1041136\_contig\_number\_19508SRS1041145\_contig\_number\_contig-100\_456.196474SRS1041157\_contig\_number\_contig-100\_517.55886SRS1041164\_contig\_number\_6756SRS104327\_contig\_number\_5368SRS1054691\_contig\_number\_20554SRS1055022\_contig\_number\_contig-100\_1545.146748SRS1055069\_contig\_number\_800SRS1055076\_contig\_number\_3640SRS1055083\_contig\_number\_8504SRS142542\_contig\_number\_21522SRS142890\_contig\_number\_9005SRS143342\_contig\_number\_21851SRS143417\_contig\_number\_contig-100\_432.137518SRS143466\_contig\_number\_2057SRS143895\_contig\_number\_38476SRS144183\_contig\_number\_42910SRS144506\_contig\_number\_50141SRS146812\_contig\_number\_56847SRS146813\_contig\_number\_contig-100\_671.153627SRS147346\_contig\_number\_50481SRS147557\_contig\_number\_33891SRS147653\_contig\_number\_contig-100\_11.59178SRS148253\_contig\_number\_3998SRS148817\_contig\_number\_9096SRS150029\_contig\_number\_20453SRS893295\_contig\_number\_11746SRS893300\_contig\_number\_contig-100\_1292.71851SRS893327\_contig\_number\_10005SRS893358\_contig\_number\_contig-100\_592.77539SRS893369\_contig\_number\_12348
species
214856
95

13
2005525
family

375288
genus
13

3
823
species

SRS018049\_contig\_number\_492SRS052078\_contig\_number\_contig-100\_1120.37867SRS101268\_contig\_number\_1107

10
46503
species

SRS014235\_contig\_number\_33897SRS019286\_contig\_number\_3340SRS055966\_contig\_number\_contig-100\_328.28918SRS056519\_contig\_number\_14188SRS062427\_contig\_number\_4893SRS063127\_contig\_number\_1699SRS142923\_contig\_number\_13123SRS147139\_contig\_number\_35589SRS148424\_contig\_number\_20580SRS893327\_contig\_number\_160
